# Laryngeal Schwannoma, Alarming Mass of Airway: A Case Report

**DOI:** 10.31729/jnma.6345

**Published:** 2021-11-30

**Authors:** Anup Samel, Shankar Prasad Shah, Shyam Thapa Chhetri, Sudip Mishra, Ashik Rajak, Prakash Banjade

**Affiliations:** 1 Department of Otorhinolaryngology & Head and Neck Surgery, BP Koirala Institute of Health Sciences, Dharan, Nepal; 2 Department of Otorhinolaryngology & Head and Neck Surgery, Kathmandu Medical College Teaching Hospital, Kathmandu, Nepal; 3 Department of Emergency Medical Services, Government of Maldives, Ministry of Health Male, Maldives

**Keywords:** *benign neoplasm*, *case report*, *larynx*, *schwannoma*, *tracheotomy*

## Abstract

Laryngeal schwannomas are rare tumors of neural sheath origin. They normally present as a slow-growing, encapsulated, submucosal mass commonly in the supraglottic region. We describe a 13-year-old boy presenting with a 4-month history of progressive worsening dysphagia. Fiber optic laryngoscopy and computed tomography revealed a polypoidal mass in the laryngeal surface of epiglottis abutting left the aryepiglottic fold, base of the tongue and hypopharyngeal wall. Direct laryngoscopic evaluation and microdebrider assisted debulking was performed with tracheostomy. Schwannoma was confirmed by histopathological study. In a regular follow-up after two months, 70 degree endoscopic evaluation revealed similar mass in the left aryepiglottic fold obscuring the vocal cord. Definite complete excision of the tumor was planned and endoscopic excision of the mass was performed with removal of ipsilateral aryepiglottic fold, arytenoid and false vocal cord with retracheotomy. Rapid occurrence of mass after debulking and biopsy was demonstrated in this case. Though rare, neurogenic tumors of the larynx are life-threatening and need complete removal.

## INTRODUCTION

Schwannoma (neurinoma/neurilemmoma) is a benign, encapsulated, slow growing submucosal tumor of nerve sheath origin first described in 1908 by Verocay^1^ and can occur anywhere along somatic or sympathetic nerve except the olfactory and optic nerves, which lack Schwann cell sheaths.^2^ Neurogenic tumors of the larynx represent less than 1.5% of all benign tumors, 3 more common in females in the 5th decade. Risk factors are radiation exposure and genetic predisposition.^4^ Suchanck described the first case in 1925 and 130 cases have been reported so far.^5^ In the larynx the tumor arises from the internal branch of the superior laryngeal nerve.^3^ Nagato described schwannoma originating from anastomosis of the internal branch of the superior laryngeal nerve and the recurrent laryngeal nerve.^6,7^ The most common anatomical site of origin is the aryepiglottic fold and malignant transformation is rare.^8^

## CASE REPORT

A 13 years old boy presented to the outpatient clinic with progressive odynophagia and exertional dyspnea over a period of four months. There were no associated systemic symptoms. He was a non-smoker and nonalcoholic. On examination, he was not dysphonic, dyspneic, or in distress. On oral examination, a reddish fleshy mass was visible in the lingual surface of the epiglottis. Indirect laryngoscopy revealed the mass obscuring the whole of the laryngeal vestibule. Flexible nasoendoscopy revealed a soft tissue swelling in the supraglottic region starting from the epiglottis blocking the view of the bilateral vocal cords. Computer tomography (CT) showed a well-defined heterogeneously enhancing polypoidal hypodense lesion extending from lower border of C2 to upper border of C5 vertebra involving laryngeal surface of epiglottis causing compression and abutting left the aryepiglottic fold, anteriorly abutting the base of the tongue and extending to the oropharyngeal lumen, posteriorly and laterally abutting supraglottic laryngeal /hypopharyngeal wall and left laterally abutting cornua of hyoid bone, medially causing narrowing of pharyngeal mucosal space and posteriorly abutting the retropharyngeal space. Tracheotomy was done beforehand and direct laryngoscopic evaluation and biopsy were performed. Intraoperatively, a solid, wellencapsulated lesion attached with the epiglottis and left aryepiglottic fold was shelled out using microdebrider and hemostasis achieved with bipolar cautery (Figures 1-5). Decannulation was done and discharged on 8th post-operative day. Histopathological examination confirmed the presence of an arytenoid schwannoma. Three months following surgery, recurrence was found in the aryepiglottic fold. Resurgery with tracheotomy and excision of mass was carried out. The child was discharged after decannulation on 5th post-operative day and kept in close follow up monthly. Endoscopic evaluation of larynx showed larynx to be completely normal clinically and the child was free of all symptoms.

**Figure 1a:**
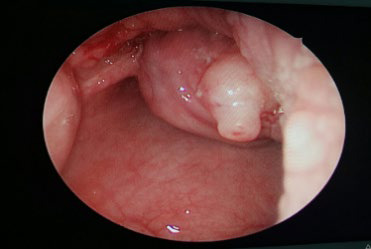
Laryngeal mass in the laryngeal

**Figure 1b:**
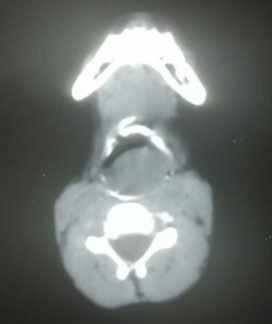
CT imaging mass during the first visit. showing a hyperdense inlet.

**Figure 2:**
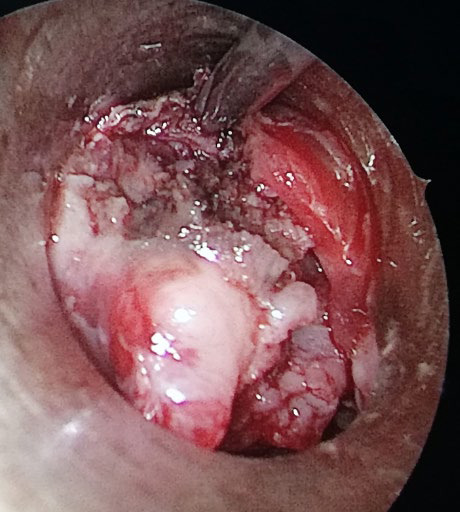
Debulking of mass and biopsy.

**Figure 3:**
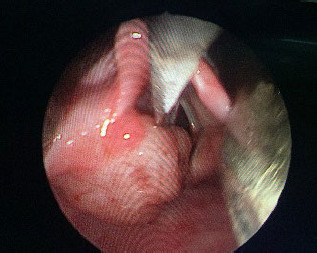
Recurrent mass in left laryngeal inlet.

**Figure 4a:**
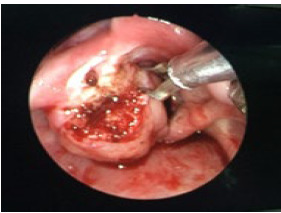
Removal of recurrent mass via microdebrider.

**Figure 4a:**
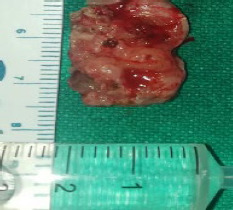
Excised mass.

**Figure 5:**
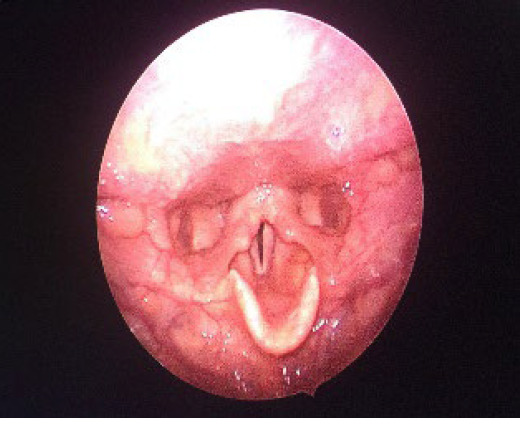
Normal appearing larynx in follow-up.

## DISCUSSION

It is important to differentiate a schwannoma from a neurofibroma as neurofibromas have a higher potential of recurrence and malignancy (approximately 10%). In addition, the surgeon should rule out neurofibromatosis in a case of neurofibroma. Histopathological examination aids definitive diagnosis. Most of the patients present with altered voice, stridor, dyspnea, dysphagia, globus sensation, or lateral neck lump. On laryngoscopy, most lesions appear as a smooth submucosal lesion, usually confined to the aryepiglottic fold or false vocal cord obstructing the view of the laryngeal inlet and resulting in reduced mobility of the vocal cord. The latter may be due to "pseudofixation" of the cricoarytenoid joint as a result of the mass effect of the lesion.^9^ Almost all the schwannoma arise from supraglottis.^10^ CT and MRI are valuable in defining the nature and extent of the lesion, with MRI offering superior soft tissue delineation. Typically, the lesion is sharply demarcated, round or oval, isoattenuated with muscle, and often heterogeneously enhanced.^11^ Calcification which reflects degenerative change, though rare, has been reported in ancient schwannomas. Schwannoma and neurofibromas cannot be distinguished radiologically due to similar findings. The differential diagnosis includes laryngeal cyst and internal laryngocele. The definitive diagnosis is performed histologically. Enger and Weiss established three histological criteria for the diagnosis of schwannoma: encapsulation, presence of Antoni A and/or Antoni B stroma, and S-100 protein positivity.^12^ In Antoni A (cellular region), the spindleshaped Schwann cells are compactly arranged with nuclei occasionally lining up in palisades to form Verocay bodies. Antoni B (less cellular) describe loosely arranged spindle Schwann cells within a myxoid matrix.

In contrast, neurofibromas are unencapsulated and comprise of a variety of cell types: elongated spindle Schwann cells interwoven with axons and collagen fibres. An important feature is that schwannoma grows extrinsic to the nerve fiber whilst in neurofibroma, the tumor is entwined with the parental nerve fascicles.^5^ Surgery forms the mainstay of treatment of laryngeal schwannoma. Schwannomas are radioresistant and hence do not respond to radiotherapy.^13^ A tracheostomy may be required to secure the airway. The surgical approach depends on the size and location of the lesion. Smaller lesions can be approached endoscopically with or without a laser. Larger tumors may require an external approach, for example, lateral thyrotomy, lateral pharyngotomy, or laryngofissure technique.^14^ Wide excision is necessary to prevent a recurrence. Rapid regrowth can occur within months following incomplete resection of the schwannoma.^5^ Following surgery, restoration of vocal cord mobility has been reported independent of the approach. In general, small tumors can be excised transorally or endoscopically, whereas larger tumors may require an open approach. Tumors located in the hypopharynx (piriform sinus, subglottis) may not be suitable for endoscopic removal, as poor exposure may result in more mucosal injury.^15^ Comparison of outcome after an open approach to an endoscopic approach does not reveal any statistical difference in disease persistence. In 2015, Ueha et al have mentioned that a new approach, called the STACA (Supra-thyroid alar cartilage approach), is particularly useful for submucosal neoformations of the larynx.^16^ This approach has been shown to be efficient and less invasive, requiring an incision of the thyroid membrane and allowing an optimal exposure of the submucosal mass in the pre-epiglottic space and superior paraglottis.^16^

Despite its rarity, neurogenic tumors of the larynx need to be recognised. The primary management involves securing the airway. Despite various imaging modalities, the distinction between Schwannoma and neurofibromas can only be made histologically and complete resection of the lesion is necessary to prevent recurrence.

## References

[1] Verocay J (1908). Multiple Geschwülste als Systemerkrankung am nervösen Apparate [Internet]..

[2] Rognone E, Rossi A, Conte M, Nozza P, Tarantino V, Fibbi A (2007). Laryngeal Schwannoma in an 8-year-old boy with inspiratory dyspnea.. Head Neck..

[3] Jones SR, Myers EN, Barnes L (1984). Benign neoplasms of the larynx.. Otolaryngol Clin North Am..

[4] Romak JJ, Neel HB, Ekbom DC (2017). Laryngeal schwannoma: a case presentation and review of the Mayo Clinic experience.. J Voice..

[5] Zbaren P, Markwalder R (1999). Schwannoma of the true vocal cord.. Otolaryngol Head Neck Surg..

[6] Plantet MM, Hagay C, De Maulmont C, Mahe E, Banal A, Gentile A (1995). Laryngeal schwannomas.. Eur J Radiol.

[7] Nagato T, Katada A, Yoshizaki T, Kunibe I, Takahara M, Katayama A (2010). Laryngeal plexiform schwannoma as first symptom in a patient with neurofibromatosis type 2.. Clin Neurol Neurosurg..

[8] Nanson EM (1978). Neurilemoma of the larynx: a case study.. Head Neck Surg..

[9] Rosen FS, Pou AM, Quinn FB (2002). Obstructive supraglottic schwannoma: a case report and review of the literature.. Laryngoscope..

[10] Thomas RL (1979). Non-epithelial tumours of the larynx.. J Laryngol Otol..

[11] Lin J, Martel W (2001). Cross-sectional imaging of peripheral nerve sheath tumors: characteristic signs on CT, MR imaging, and sonography.. AJR Am J Roentgenol..

[12] Woodruff JM, Kourea HP, Louis DN, Schethauer BW (2000). WHO Classification of Tumours: Pathology and Genetics of Tumors of Nervous System..

[13] Cadoni G, Bucci G, Corina L, Scarano E, Almadori G (2000). Schwannoma of the larynx presenting with difficult swallowing.. Otolaryngol Head Neck Surg..

[14] Takumida M, Taira T, Suzuki M, Yajin K, Harada Y (1986). Neurilemmoma of the larynx: a case report.. J Laryngol Otol..

[15] Park KT, Ahn Y, Kim KH, Kwon TK (2010). Schwannoma mimicking laryngocele.. Clin Exp Otorhinolaryngol..

[16] Ueha R, Nito T, Sakamoto T, Fujimaki Y, Yamauchi A, Yamasoba T (2015). Supra-thyroid alar cartilage approach for the complete resection of laryngeal submucosal tumors and postoperative voice quality.. Eur Arch Otorhinolaryngol..

